# Dr. Gardner Byron Young

**Published:** 1918-12

**Authors:** 


**Dr. Gardner Byron Young,** Bellevue 1886, died at his home in Geneva, Oct. 1, aged 58.

